# Public Preferences for Social Distancing Policy Measures to Mitigate the Spread of COVID-19 in Missouri

**DOI:** 10.1001/jamanetworkopen.2021.16113

**Published:** 2021-07-08

**Authors:** Ingrid Eshun-Wilson, Aaloke Mody, Virginia McKay, Matifadza Hlatshwayo, Cory Bradley, Vetta Thompson, David V. Glidden, Elvin H. Geng

**Affiliations:** 1 School of Medicine, Division of Infectious Diseases, Washington University in St Louis, St Louis, Missouri; 2 Brown School at Washington University in St. Louis, St Louis, Missouri; 3 Center for Dissemination and Implementation Research, Washington University in St Louis, St Louis, Missouri; 4 School of Medicine, University of California, San Francisco

## Abstract

**Importance:**

Policies to promote social distancing can minimize COVID-19 transmission but come with substantial social and economic costs. Quantifying relative preferences among the public for such practices can inform locally relevant policy prioritization and optimize uptake.

**Objective:**

To evaluate relative utilities (ie, preferences) for COVID-19 pandemic social distancing strategies against the hypothetical risk of acquiring COVID-19 and anticipated income loss.

**Design, Setting, and Participants:**

This survey study recruited individuals living in the Missouri area from May to June 2020 via randomly distributed unincentivized social media advertisements and local recruitment platforms for members of minority racial and ethnic groups. Participants answered 6 questions that asked them to choose between 2 hypothetical counties where business closures, social distancing policy duration, COVID-19 infection risk, and income loss varied.

**Main Outcomes and Measures:**

Reweighted population-level relative preferences (utilities) for social distancing policies, subgroups, and latent classes.

**Results:**

The survey had a 3% response rate (3045 of 90 320). Of the 2428 respondents who completed the survey, 1669 (75%) were 35 years and older, 1536 (69%) were women, and 1973 (89%) were White. After reweighting to match Missouri population demographic characteristics, the strongest preference was for the prohibition of large gatherings (mean preference, −1.43; 95% CI, −1.67 to −1.18), with relative indifference to the closure of social and lifestyle venues (mean preference, 0.05; 95% CI, −0.08 to 0.17). There were weak preferences to keep outdoor venues (mean preference, 0.50; 95% CI, 0.39 to 0.61) and schools (mean preference, 0.18; 95% CI, 0.05 to 0.30) open. Latent class analysis revealed 4 distinct preference phenotypes in the population: risk averse (48.9%), conflicted (22.5%), prosocial (14.9%), and back to normal (13.7%), with men twice as likely as women to belong to the back to normal group than the risk averse group (relative risk ratio, 2.19; 95% CI, 1.54 to 3.12).

**Conclusions And relevance:**

In this survey study using a discrete choice experiment, public health policies that prohibited large gatherings, as well as those that closed social and lifestyle venues, appeared to be acceptable to the public. During policy implementation, these activities should be prioritized for first-phase closures. These findings suggest that policy messages that address preference heterogeneity (eg, focusing on specific preference subgroups or targeting men) could improve adherence to social distancing measures for COVID-19 and future pandemics.

## Key Points

**Question:**

What are public preferences for social distancing measures to mitigate COVID-19 transmission in a typical midwestern US state?

**Findings:**

In this survey study with 2428 participants, most respondents were willing to tolerate the prohibition of large gatherings and closure of social and lifestyle venues. However, acceptable trade-offs varied, and distinct preference subgroups were identified.

**Meaning:**

The findings suggest that social distancing policies that prohibit large gatherings and close social and lifestyle venues would be well aligned with public preferences, but public health campaigns will need to develop targeted strategies to improve acceptability and adherence in specific subgroups.

## Teaser

This survey study evaluates public preferences for social distancing strategies weighed against the hypothetical risk of acquiring COVID-19 and anticipated income loss.

## Introduction

Nonpharmaceutical means of stemming the COVID-19 pandemic were a necessary component of the public health response throughout the United States during the last year and, in many parts of the world, may remain a consideration for the foreseeable future. However, many of these practices carry formidable economic and social costs, giving rise to complicated considerations about potential benefits and harms for individuals as well as society at large. In the setting of at least some uncertainty, how individuals weigh the desirability and harms of such policies can help policy design meet public preferences when possible. Behavioral science has found that designing programs to be easy, attractive, and aligned with population preferences can have important effects on behavior. Quantifying such preferences, how they group, and associations with sociodemographic factors can inform policy design for COVID-19 as well as future pandemics and disasters.

Information about public perspectives and attitudes toward social distancing policies have been prominent in the lay press, but formal research evidence about preferences is not widely available. For example, the public’s attention has been drawn toward high-profile instances in which vociferous opposition to social distancing policies led to threats against public health officials. However, the prevalence and strength of these beliefs may be overrepresented in media. The research that does exist to date indicates general support for social distancing policies.^1,2^ However, these studies do not capture the relative desirability (or undesirability) of different types of distancing policies, nor do they capture the public’s willingness to trade between different preferences. Without such data, information for setting priorities, when no single solution will be sufficient and all come with costs and harms, is incomplete. In addition, the significant heterogeneity of social distancing policy implementation and adherence across the United States means that there is a need for locally relevant data.

In this study, we used a discrete choice survey (also known as a discrete choice experiment [DCE] or conjoint analysis), which is widely used in marketing. We applied this technique to examine preferences for social distancing measures in Missouri, a state that is demographically and economically representative the US Midwest region.

## Methods

A DCE is a survey design that solicits utilities from respondents.^3^ Utilities have been defined as happiness or preferences, and the concept comes from economic theory. By alternating the features (levels) of a set of service, product, or policy attributes, a DCE can quantify relative utilities (ie, mean preferences) for any of the features. The study was approved by the Washington University institutional review board and was exempted from a formal consent process because data collection was anonymous; instead, participants were provided with an introductory information sheet as part of the online survey. We adhered to guidelines as set forth by the International Society for Pharmacoeconomics and Outcomes Research (ISPOR) reporting guideline for design and reporting of the research question, survey attributes and levels, task construction, instrument design and statistical analysis.^4^

### Study Population

To draw from the state of Missouri broadly, the survey was distributed using social media advertising on Facebook and Instagram. These platforms sent advertisements to a randomly selected subset of users in the Missouri area daily for 23 days. We also distributed the survey through the Center for Community Health Partnership and Research at the Institute for Public Health at Washington University in St Louis, using existing social media networks. The survey was fielded between May 21 and June 13, 2020 (eFigure 1 in the Supplement). No incentive was provided to participants.

### Survey and DCE Design

The survey solicited several possible effect modifiers of preferences including age, gender, race, annual household income, and chronic health conditions for respondents. Racial categories (ie, American Indian or Alaska Native, Asian, Black or African American, Native Hawaiian or other Pacific Islander, White, other, and prefer not to answer) were based on reduced categories from the US census surveys. Because there is clear evidence that racial minority populations have been disproportionately affected by COVID-19, it follows that preferences may vary by race. Selection of social distancing policy features for inclusion in the DCE was informed by literature review and consultation with local experts in infectious diseases, social sciences, and public health and by DCE design guidelines^5,6,7,8,9^ (eMethods in the Supplement). This resulted in the selection of 7 attributes as follows: (1) closure of educational facilities, (2) closure of indoor social and lifestyle services (eg, salons, bars), (3) closure of outdoor recreation services (eg, parks, beaches), (4) prohibition of large gatherings (eg, large conferences, religious events), (5) duration of the policy, (6) risk of infection for the individual, and (7) potential income loss. In the DCE, participants were asked to choose between 2 hypothetical counties with different policies, COVID-19 risk levels, and income attribute levels, all else being equal (Table 1).^4,10^

**Table 1.** Attributes and Levels Included in Discrete Choice Experiment Survey^a^

Attribute	Levels
Duration of policy	1 mo 2 mo 3 mo
Income lost in 6 months, %	5 15 25
Educational facilities (eg, childcare, schools, colleges)	Open Closed
Outdoor activity venues (eg, national parks, beaches)	Open Closed
Large gatherings (eg, conferences, sports, religious events)	Permitted Not permitted
Social and lifestyle venues (eg, restaurants, bars, salons, gyms)	Open Closed
Risk of COVID-19 infection in 6 mo	Low; 5% or 1 in 20 chance Moderate; 10% or 1 in 10 chance High; 30%: or 3 in 10 chance

^a^ The discrete choice question was as follows: “In a hypothetical situation (a situation that does not necessarily exist in real life), where two counties have different social distancing policies and consequences, and you could choose to live in either, which of these two counties would you choose to live in?”

We used a near-balanced (ie, all attribute levels appear an equal number of times across the experiment) and near-orthogonal (ie, each attribute pair appears an equal number of times across the experiment) fractional factorial design. We used the logit efficiency test to evaluate design efficiency against a simulated data set to ensure adequate precision (standard errors, <0.05). We generated 300 versions of the choice experiment and randomly ordered the position of the attributes. We used Lighthouse Studio version 9.10.1 (Sawtooth Software) for design and data collection.

### Statistical Analysis

We used mixed logit regression models to estimate mean preferences (ie, relative utilities) in the overall population. For all analyses, we weighted responses using inverse probability weights to represent the target population of Missouri by age, gender, and race^11,12^ (eTable 1 in the Supplement). To explore preference heterogeneity in the population, we conducted subgroup analyses by sociodemographic characteristics, such as gender, annual household income, age, chronic health condition, and race group. We also conducted latent class analysis to identify preference groupings in the responses themselves. To do so, we fit latent class conditional logit models and used model fit criterion (Akaike and bayesian information criterion) as well as qualitative exploration to determine the optimum number of latent classes. We validated latent class membership using cross-validation techniques.^13^ We evaluated factors associated with membership in identified latent classes using multinomial logistic regression models. To determine marginal probabilities of belonging to 1 of 2 specific latent classes according to demographic characteristics, we used generalized linear models with a log-link function. To quantify trade-offs in preferences, we additionally conducted willingness-to-trade analyses for social distancing policies (against the percentage of income lost or risk of COVID infection in the county) overall and within subgroups. We calculated trade-offs using nonlinear combinations of estimators to determine which combination of attribute utilities were equivalent to percentage income loss or infection risk and present the difference in mean preferences and 95% CIs. We used Stata version 16 (StataCorp) to conduct analyses, and all significance tests were 2-tailed and at the 95% level of significance.

## Results

Of the 3045 people who clicked on the survey link (of 90 320, for a 3% response rate), 2428 respondents completed the survey. Of these, 1536 (69%) were female individuals, 1973 (89%) were White individuals, and most (1739 [78%]) had an annual household income greater than $50 000 and were 35 years or older (1669 [75%]). Overall, 751 respondents (31%) reported chronic health conditions (Table 2). The 617 respondents who did not complete the survey were similar to those who did complete it with regard to gender and race distribution; however, those who did not complete the survey appeared to be older (eTable 2 in the Supplement).

**Table 2.** Demographic Characteristics of Participants

Characteristic	Respondent, No. (%) (N = 2428)
Age, y	
18-24	126 (6)
25-34	424 (19)
35-49	553 (25)
50-64	647 (29)
≥65	469 (21)
Gender	
Male	667 (30)
Female	1536 (69)
Nonconforming or other	12 (1)
No answer	4 (<1)
Race	
Black	127 (6)
White	1973 (89)
Other^a^	92 (4)
No answer	27 (1)
Chronic health conditions^b^	
No chronic health conditions	1535 (69)
Respiratory chronic health conditions	320 (14)
Other chronic health conditions	431 (19)
No answer	11 (<1)
Annual household income, $	
<20 000	97 (4)
20 000-49 000	383 (17)
50 000-99 000	871 (39)
≥100 000	868 (39)
No answer	209 (9)

^a^ Other includes individuals who responded American Indian or Alaska Native, Asian, Native Hawaiian or other Pacific Islander, other, and prefer not to answer.

^b^ Chronic health conditions were not mutually exclusive. Other chronic health conditions included diabetes, chronic kidney disease, immunosuppressive disorders, and cancer.

### Main Preferences

**Figure 1.**
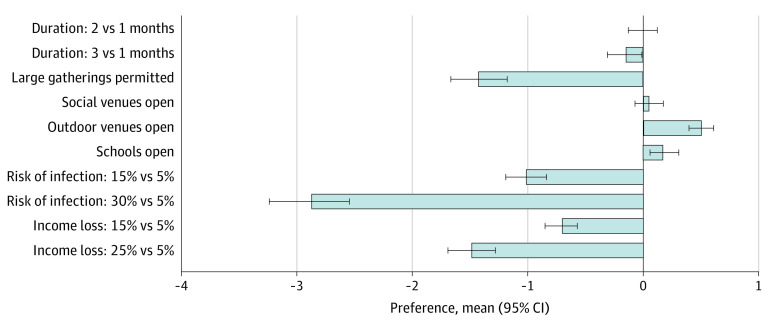
Mean Preferences for Social Distancing Measures in the Population

The strongest mean preferences for social distancing policy measures were for large gatherings to be prohibited (permitted vs prohibited: mean preference, −1.43; 95% CI, −1.67 to −1.18), followed by a preference for keeping outdoor recreational venues open (open vs closed: mean preference, 0.50; 95% CI, 0.39 to 0.61). Weaker preferences were observed for keeping educational facilities open (open vs closed: mean preference, 0.18; 95% CI, 0.05 to 0.30) and shorter durations of the social distancing policy (3 months vs 1 month: mean preference, −0.16; 95% CI, −0.31 to −0.02). Participants did not show any specific preference for keeping social and lifestyle venues open (open vs closed: mean preference, 0.05; 95% CI, −0.08 to 0.17) (Figure 1; eTable 3A and 3B in the Supplement). There was substantial preference heterogeneity for several attributes, as evidenced by large SDs in relation to the relative utilities generated in mixed logit models, for prohibiting large gatherings (SD, 2.62; 95% CI, 2.14 to 3.09), keeping social venues open (SD, 1.01; 95% CI, 0.76 to 1.27), and keeping schools open (SD, 1.13; 95% CI, 0.85 to 1.14). We explored this preference heterogeneity through subgroup and latent class analysis. Preferences across subgroups largely mirrored main preferences; there were no substantial differences between demographic subgroups across analyses (eFigure 2 and eTable 4 in the Supplement).

### Latent Class Analysis

**Figure 2.**
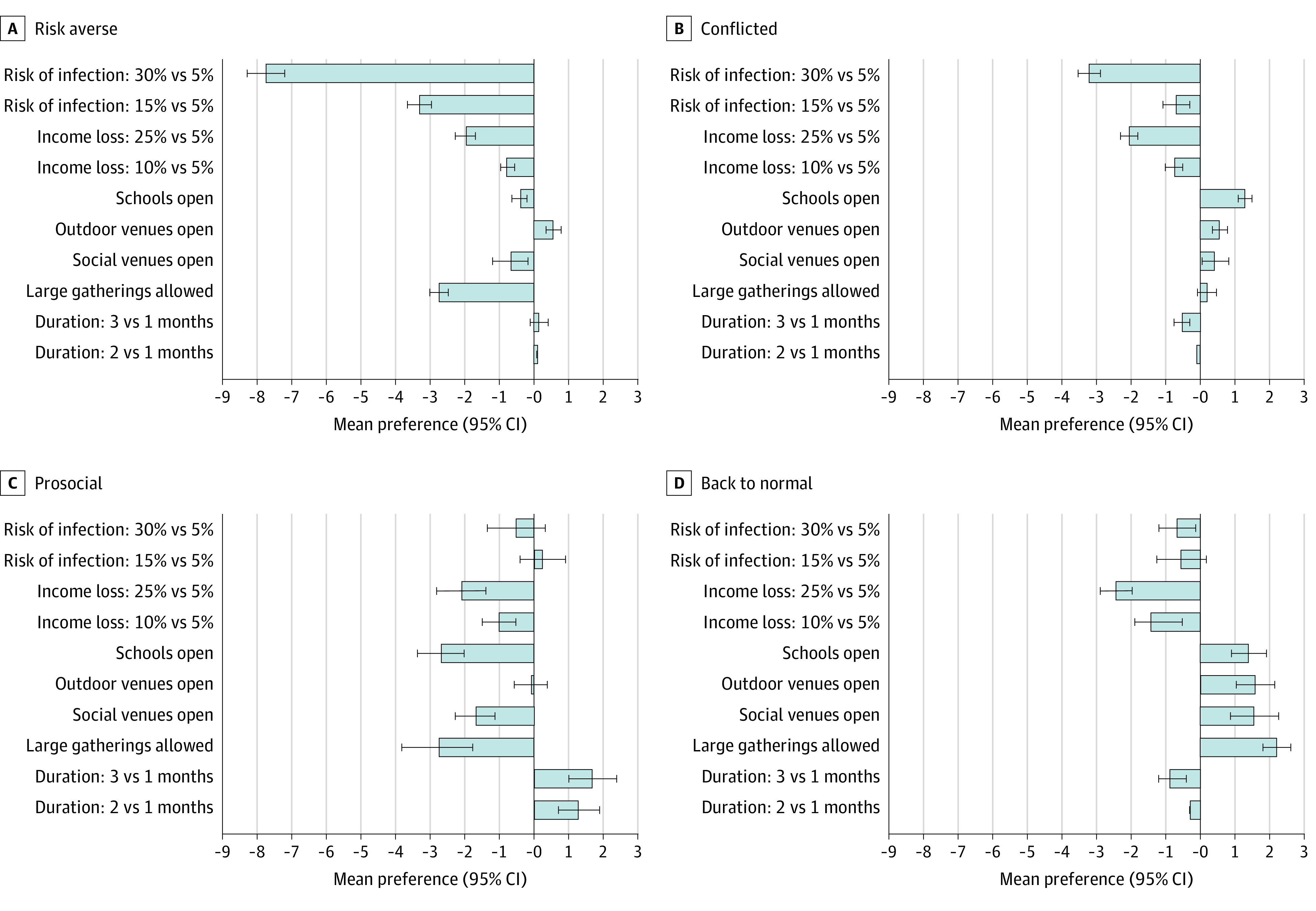
Mean Preferences for Social Distancing Measures in the Population Across 4 Latent Class Preference Groups

Four latent preference classes were identified (Figure 2; eTable 5 in the Supplement). The largest group (48.9%), the risk averse, had strong preferences for all possible restrictive policy options (eg, mean preference for permitting large gatherings, −2.78; 95% CI, −3.30 to −2.27) (Figure 2A). A second conflicted group (22.5%) showed a preference for minimizing risk but keeping schools open (mean preference for keeping schools open: 0.46; 95% CI, 0.26 to 0.66) (Figure 2B). A third, prosocial group (14.9%), while strongly preferring that all services remain closed (eg, mean preference for keeping schools open: −2.71; 95% CI, −3.39 to −2.04), showed mild preferences for reducing their personal risk of infection (Figure 2C). The fourth, back to normal group (13.7%) showed a strong preference for keeping all services open (mean preference for large gatherings permitted: 2.19; 95% CI, 1.50 to 2.87), with a relatively weak overall preference for reducing their infection risk (Figure 2D). Male gender was strongly associated with the back to normal group compared with the risk averse group (relative risk ratio, 2.19; 95% CI, 1.54-3.12) (eTable 6 in the Supplement). Marginal estimates showed this gender differential trend across strata of income, race, chronic health conditions, and age (except among those older than 65 years) (Table 3).

**Table 3.** Marginal Probabilities of Belonging to Back to Normal Group vs Risk Averse Group by Gender

Characteristic	Marginal probability of belonging to back to normal vs risk averse latent class group, % (95% CI)	
	Female respondent	Male respondent
Age, y		
18-24	13.2 (3.8-22.5)	28.1 (9.0-47.2)
25-34	9.3 (5.3-13.3)	18.3 (9.6-26.9)
35-49	12.8 (8.9-16.7)	23.1 (13.7-32.6)
50-64	14.3 (9.7-18.9)	34.9 (22.5-47.2)
≥65	13.4 (6.5-20.3)	14.1 (6.8-21.3)
Race		
Black or African American	2.9 (0-6.3)	30.1 (0-70.0)
White	14.0 (11.5-16.5)	23.5 (18.8-28.1)
Other^a^	12.2 (0-28.4)	29.5 (8.4-50.6)
Annual household income, $		
<50 000	9.8 (5.0-14.7)	28.5 (14.8-42.3)
50 000-99 999	13.5 (9.2-17.8)	24.1 (15.7-32.4)
≥100 000	13.5 (9.9-17.0)	21.7 (14.6-28.7)
Chronic health condition		
Yes	14.6 (11.5-17.8)	24.8 (18.4-31.2)
No	8.0 (4.7-11.3)	22.6 (13.2-31.9)

^a^ Other includes individuals who responded American Indian or Alaska Native, Asian, Native Hawaiian or other Pacific Islander, other, and prefer not to answer.

In an analysis of trade-offs within latent class groups, those who were risk averse were willing to keep all services closed for a period of 3 months, give up 25% of their income, and potentially tolerate more restrictions to live in a county with a 5% rather than a 30% risk of infection (utility difference, −1.46; 95% CI, −2.44 to −0.47, *P* = .004). In contrast, the back to normal group was willing to tolerate a 30% vs 5% risk of infection (utility difference, −0.86; 95% CI, −2.07 to 0.36; *P* = .17) to prevent losing 25% of their income and to keep all services open for a period of 3 months.

## Discussion

Most respondents in this study were concerned about their COVID-19 infection risk and were willing to give up a number of social freedoms and services as well as a moderate percentage of their income to reduce this risk. Preferences indicated a strong aversion to the acquisition of COVID-19, which in the ranges of risk offered in this survey, outweighed countervailing considerations, such as the closure of social venues and loss of income. Latent class analysis revealed 4 distinct groups defined by different patterns of preferences. The largest group (ie, risk averse), comprising approximately half of the population, were highly disinclined to acquiring COVID-19 and had minimal desire for normal social activities. The second group—the conflicted, representing nearly one-quarter of respondents—also displayed a strong aversion to infection but, at the same time, expressed strong desires to see venues, schools, and business open. The third group, which we called the prosocial group (including approximately 1 in 6 individuals) professed relatively weak aversion to infection but also little desire to see normal social and economic activity return immediately. Finally, the back to normal group (also approximately 1 in 6 persons) displayed surprisingly little aversion to infection but instead had strong preferences to see normal social function, opening of restaurants and businesses, and resumption of large gatherings. Of note, membership in these classes was not strongly associated with sociodemographic factors, although male sex seemed to predispose individuals to membership in the back to normal group. These data mirror cross-sectional surveys supporting stay-at-home orders and nonessential business closures,^2^ extend existing data by dissecting the elements of social distancing that drive overall attitudes, and uncover preference segments in the population that do not fall between easily observed sociodemographic lines.

Segmentation of preferences for social distancing practices provides deeper insights about how the US public in Missouri has reacted to the COVID-19 epidemic and the public health response. First, a large segment of the population evinced a strong aversion to risk of infection and considered normal social function of little importance. Two preference subgroups in this data (the risk averse and prosocial groups) were driven either by concerns of becoming infected or a desire to reduce transmission in the community and abide by public health guidance, likely representing those who will comply with social distancing measures.^14^ These groups had particularly strong preferences for the prohibition of large gatherings, likely influenced by widespread media reporting of so-called superspreader events that have fueled the COVID-19 pandemic within and outside the US and a growing understanding of which situations pose the highest risk of COVID-19 transmission.^15^ Furthermore, while visiting social and lifestyle venues, such as restaurants, bars, gyms, and hair salons, may have been important to participants, these groups were more willing to give up these activities to reduce their COVID-19 infection risk than give up education and outdoor recreation, potentially supporting a phased approach to social distancing policy implementation in the future, in which large gatherings and social and lifestyle venues are closed as a first step.

The remaining population was more willing to make trade-offs with infection risk to keep services open. The conflicted group was willing to accept some infection risk to keep a few services open, specifically schools. School closures have been a topic of debate, with uncertainty regarding the impact of school closures on reducing COVID-19 transmission and mortality, which is reflected in population preferences in these data.^16^ The back to normal group, who were twice as likely to be men as women, primarily focused on preserving income; keeping all social, lifestyle, and educational services open; and permitting large gatherings, such as conferences and sports events, with relatively less concern for COVID-19 infection risk. Across strata of age, race, income, and comorbid illness, men were more likely to fall into this group than women. A gender differential in COVID-19 risk perception and social distancing adherence has been identified in several surveys, including cross-sectional data from more than 20 000 participants across 8 countries, with women more likely to comply with public health measures to prevent COVID-19 infection and to perceive COVID-19 as a very serious health problem.^1,2,17^ However, there was still a proportion of women who belonged to this back to normal group, suggesting further drivers of subgroup membership. In the United States, compliance with COVID-19 preventative measures has been highly politicized,^18,19,20^ and partisanship may have additionally informed preferences and membership in this group.

This DCE also offers insights into particular behavioral phenotypes, identified in randomized trials of vaccine messaging and earlier behavioral research, and how these might segment in a population. For example, the prosocial impulse may have underpinned the preferences of those for whom concerns of the harms of the pandemic on society outweighed individual concerns.^14^ Furthermore, vaccine messaging incorporating the message of getting back to normal appeared to be effective, which aligned with our observation that a return to normality is highly desired.^21^ Each of these behavioral phenotypes point toward social and psychological groupings in society that are important to surface because they are not easily identified by sociodemographic characteristics but are likely to drive behavior.

### Limitations

This study has a number of limitations. DCE’s represent hypothetical situations that may not reflect how individuals make choices in real life, especially when low income, financial vulnerability, and the absence of social supports and safety nets mean that people are unable to align behavior with preferences. However, several of our findings are reflected in other cross-sectional surveys.^1,2,17^ We did not include mask wearing as an attribute in the DCE. The politicization of mask wearing may have resulted in this attribute dominating others in the choice experiment. Data from other settings show that adherence to mask wearing is well aligned with adherence to other social distancing measures.^18^ Recruitment of study participants through social media tools can result in a cohort with predominantly affluent female study participants, whose preferences may not be representative of inference populations.^22^ To account for this, we applied population inverse probability sampling weights to ensure that preferences reflected the demographic structure of Missouri. The attribute related to income loss did not specify personal income loss, and as a result, it is possible that some responded to this as income lost by the community. We also did not collect data on previous COVID-19 infection. At the time of the survey, the prevalence of known diagnosis in the region was considerably less than 5%; however, it is possible that the preferences of those who had had COVID-19 vs those who had not may have differed. Deploying the DCE during a pandemic may also have resulted in participants being unable to truly see scenarios as hypothetical (as required by the choice tasks). Given that this DCE was conducted early in the COVID-19 pandemic, preferences and tolerance for service closures may have changed over time.

## Conclusions

In this study, prohibiting large gatherings and closing social and lifestyle venues appeared to be acceptable to the public. When public health departments face difficult decisions regarding which social distancing measures to institute, preference data such as these can help guide decision-making. In this setting, it appeared that a tiered approach that prohibits large gatherings and closes nonessential indoor social and lifestyle businesses before closing schools or outdoor facilities would be most acceptable. This, combined with targeted public health messaging addressing preference heterogeneity (eg, targeted at men or specific preference phenotypes), may further improve adherence to social distancing measures.

## Notes

Supplement. **eMethods.** Study Design and Recruitment **eFigure 1.** Example of DCE Survey Tool **eFigure 2. **Mean Preferences by Subgroup **eTable 1. **Weighting Strategy **eTable 2. **Characteristics of Those Who Did Not Complete Survey **eTable 3.** Mean Preferences and Main Model Selection **eTable 4.** Subgroup Analyses Mean Preferences **eTable 5.** Latent Class Mean Preferences and Model Selection **eTable 6.** Factors Associated With Latent Class Membership eReferences.
