# Supplementary material for: Public Preferences for Social Distancing Policy Measures to Mitigate the Spread of COVID-19 in Missouri
Source: JAMA Netw Open. 2021 Jul 8;4(7):e2116113. doi: 10.1001/jamanetworkopen.2021.16113 (PMC8267603; doi:10.1001/jamanetworkopen.2021.16113)
Supplement: Supplement. — eMethods. Study Design and Recruitment eFigure 1. Example of DCE Survey Tool eFigure 2. Mean Preferences by Subgroup eTable 1. Weighting Strategy eTable 2. Characteristics of Those Who Did Not Complete Survey eTable 3. Mean Preferences and Main Model Selection eTable 4. Subgroup Analyses Mean Preferences eTable 5. Latent Class Mean Preferences and Model Selection eTable 6. Factors Associated With Latent Class Membership eReferences. [file jamanetwopen-e2116113-s001.pdf]

Supplemental Online Content

Eshun-Wilson I, Mody A, McKay V, et al. Public preferences for social distancing policy measures to mitigate the spread of COVID-19 in Missouri. *JAMA Netw Open.* 2021;4(7):e2116113. doi:10.1001/jamanetworkopen.2021.16113

eMethods. Study Design and Recruitment

eFigure 1. Example of DCE Survey Tool

eFigure 2. Mean Preferences by Subgroup

eTable 1. Weighting Strategy

eTable 2. Characteristics of Those Who Did Not Complete Survey

eTable 3. Mean Preferences and Main Model Selection

eTable 4. Subgroup Analyses Mean Preferences

eTable 5. Latent Class Mean Preferences and Model Selection

eTable 6. Factors Associated With Latent Class Membership

eReferences.

This supplemental material has been provided by the authors to give readers additional information about their work.

© 2021 Eshun-Wilson I et al. *JAMA Network Open.*

eMethods. Study Design and Recruitment

Attribute selection:

We sought to identify attributes that were unconfounded, which is to say unlikely to be both representations of an underlying common but unsolicited preference, as well as present participants with a range of response categories that was wide enough to capture significance heterogeneity in preferences but within a range where a linear relationship was considered plausible for continuous attributes. We identified several candidate social distancing policy features of importance, including: (1) the duration of the policy, (2) the clarity of the messaging regarding the policy end date, (3) the closure of, childcare services, schools and colleges, indoor lifestyle services (e.g. salons, bars), outdoor recreation services (parks, beaches), religious services and mass gatherings. In addition, we determined that risk of infection or hospitalization for the individual and others, as well as income loss were other key determinants of adherence to social distancing public health measures. Through an iterative process of brainstorming/discussion, reducing and merging attributes to prevent overlapping concepts, reduction of number attributes to minimize cognitive burden, removal of inappropriate attributes and refinement of wording we refined attributes.

DCE Design:

In the experiment design we sought to balance pragmatism and completeness and therefore limited the number of attributes according to DCE design guidelines (five to seven attributes) and selected those attributes which we determined to be key decision drivers and of the greatest public health policy significance during the time period. To further maximize statistical and response efficiency (avoid fatigue in respondents) we limited the number of attribute levels (<=3) and the number of prohibited attribute level combinations and limited the number of DCE questions asked of each respondent to six and opted for two policy scenarios per task. We manually removed combinations considered non sensical. The final design presented consumers with two potential counties, with different sets of policies, and sought to understand which location participants preferred, all else being equal. Each policy reflected 7 attributes related to the opening or closure of social venues, education facilities and outdoor activity services, whether large gathering were permitted, the duration of the policy, the potential income lost during the first six months after the policy was instituted and the associated underlying risk of COVID infection in the county (eTable 1).

© 2021 Eshun-Wilson I et al. *JAMA Network Open.*

To achieve statistical efficiency, we constructed a near balanced (i.e., each level appears equally often across the experiment) and near orthogonal (i.e., each pair of levels across attributes appears equally) design– based on a design of 7 attributes, 4 with 2 levels and 3 with 3 levels and 6 choice sets (questions) with two scenarios each. We additionally prohibited two attribute combinations in the design – "permitted large gatherings" and "risk of COVID infection - low" and "prohibited large gatherings" and "risk of COVID infection - high". We tested the design efficiency using the logit efficiency test in Sawtooth software with simulated data to obtain an efficient design with standard errors of 0.05 or less for the main-effects analysis for the estimated sample size of 600 participants.

Sample size estimation: We based our sample size calculation on the formula $N \geq (500 \times c)/(a \times t)$ - where N is the number of participants, t is the number of choice tasks (questions), a is the number of alternative scenarios and c is the largest number of attribute levels for any one attribute, and when considering two-way interactions, 'c' is equal to the largest product of levels for any two attributes - (500 x 9/ 2 x 6) (1). To additionally conduct subgroup analyses, at least 200 participants per subgroup is recommended. The DCE was powered to detect main effects and evaluate at least 3 subgroups (minimum calculated sample size of 600). We followed the Professional Society for Health Economics and Outcomes Research (ISPOR) guidelines for design of choice experiments (2, 3).

Setting and recruitment:

The DCE was conducted in Missouri, a Mid-Western state in the US, with a population of 6,137,428. The majority of the population is white (83%) and 12% is Black/African American (4, 5). We used randomly allocated social media advertising on Facebook and Instagram to recruit participants in the state. In addition, the survey was distributed via email to study volunteer networks, and to obtain preferences of Black/African Americans the survey was distributed through targeted social media networks linked to the Washington University Center for Community Health Partnership and Research at Washington University in St. Louis. Survey fielding commenced on 21 May 2020, a period following the lifting of a state wide stay at home order - all businesses were reopened in Missouri in early May 2020 with social distancing requirements, and full restrictions were lifted on 16 June 2020. No incentive was offered to participants.

Measurements:

© 2021 Eshun-Wilson I et al. *JAMA Network Open.*

We carried out one round of cognitive interviews and piloted the final survey questions iteratively to ensure intelligibility and coherency. The survey was programmed using Sawtooth Software and participants completed the survey using personal mobile devices or computers. Participants were randomly allocated to one of 300 versions of the choice experiment and the order of the attributes within each question was randomized.

Analysis:

Mixed logit model

Choice experiment modelling is based on random utility theory (RUT) which assumes that the utility (U) for individual i conditional on choice j consists of an explainable component (Vij) and a random component (e_{ij}) (formula 1). The random component may capture any combination of unobserved attributes, unobserved preference variation, specification error, measurement error and inherent variability within and between individuals (6).

$$1. \quad U_{ij} = V_{ij} + e_{ij}$$

For this analysis we applied dummy coding. For our main effects final model we selected a mixed logit regression model to account for preference heterogeneity with all attributes included as random parameters. The explainable component (V_{ij}) for this experiment is denoted in formula 2 below, where $b_{1\text{-}10}$ represents the coefficient for the corresponding attribute level. The baseline attribute category for each attribute is omitted from formulae and estimations, as this attribute has by definition a utility of 0 when dummy coding is used.

$$2. \quad V_{ij} = b_1\,\textit{duration: 2 months} + b_2\,\textit{duration: 3months} + b_3\,\textit{income loss: 15\%} + b_4\,\textit{income loss: 25\%} + b_5\,\textit{larger gatherings: prohibited} + b_6\,\textit{social venues: open} + b_7\,\textit{outdoor venues: open} + b_8\,\textit{schools: open} + b_9\,\textit{risk of infection: 15\%} + b_{10}\,\textit{risk of infection: 30\%}$$

Mixed logit models were fit using Stata's mixlogit command which uses simulated maximum likelihood estimators and generates mean utilities for the population and standard deviations of the random coefficients (7). Mixed logit coefficients (b) can be interpreted as the strength of the relative preference for the particular attribute comparison, with positive coefficients representing positive preferences (desirable) and negative

© 2021 Eshun-Wilson I et al. *JAMA Network Open.*

coefficients representing negative preferences (less desirable). Standard deviations represent preference heterogeneity for attribute comparisons, with a 0 standard deviation indicating no heterogeneity.

Willingness to trade

We further assessed trade-offs by conducting a willingness to trade analysis which is analogous to a traditional willingness to pay analysis (8). Willingness to pay analyses routinely rely on the assumption of linearity between levels of a continuous attribute (eg. cost, waiting time), given that this assumption of linearity was unlikely to hold beyond the values presented in the experiment, we used nonlinear combinations of estimators in Stata to calculate which combination of utilities would be equivalent to the utility for 30% risk of infection versus 5% risk of infection, thereby determining what participants would be willing to trade in terms of infection risk, income, duration of policy and service closures.

Willingness to risk infection was calculated as:

$$b_{difference} = b_{10}\,\textit{risk of infection: 30\%} - (\,b_4\,\textit{income loss: 25\%} + b_2\,\textit{duration: 3months} + b_5\,\textit{large gatherings: prohibited} + b_6\,\textit{social venues: open} + b_7\,\textit{outdoor venues: open} + b_8\,\textit{schools: open})$$

Latent class analysis

We fit latent-class conditional logit models through an expectation-maximization algorithm (9). We fit up to five latent class conditional logit models using maximum likelihood estimation of datasets expanded by sampling weights and selected the model with the smallest model fit criterion (Akaike and Bayesian information criterion), the highest mean probability of group membership and the smallest number of participants with a low probability of group membership in each group. We additionally qualitatively evaluated latent classes to ensure that classes matched heterogeneity demonstrated in main and subgroup analyses. We validated latent class membership using cross-validation techniques (10).

Multinomial logit model

We applied multinomial logit regression to evaluate predictors of latent class membership. Multinomial logistic regression is conducted in the case where a dependent variable is not continuous and has more than two levels – as is the case with 4 latent classes. The model output presents the relative risk ratio which represents

© 2021 Eshun-Wilson I et al. *JAMA Network Open.*

the risk ratio between an exposure level and the baseline level for a particular exposure compared to the selected comparison group – in our case the ‘risk averse’ latent class.

Marginal probabilities

To evaluate marginal probabilities of belonging to the “back to normal” or “risk averse” group we additionally conducted as series of binary logistic regression models with these two categories as the dependent variable and fit an interaction term between gender and other demographic characteristics and generated marginal estimates based on these models, i.e. the probabilities of belonging to each latent class group by gender and demographic characteristic.

© 2021 Eshun-Wilson I et al. *JAMA Network Open.*

eFigure 1. Example of DCE Survey Tool

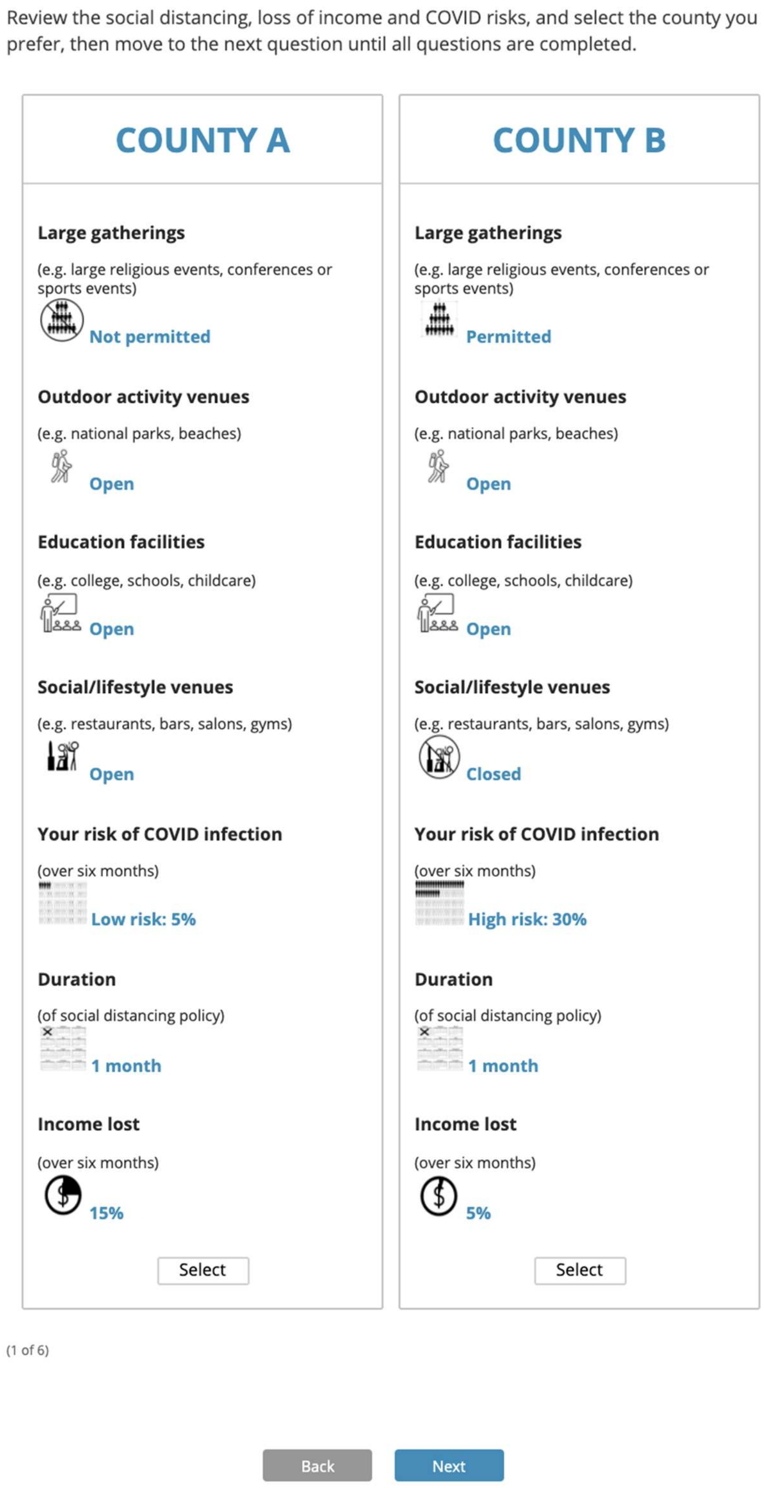

© 2021 Eshun-Wilson I et al. *JAMA Network Open.*

eFigure 2. Mean Preferences by Subgroup

eFigure 2a: Mean preferences by age category

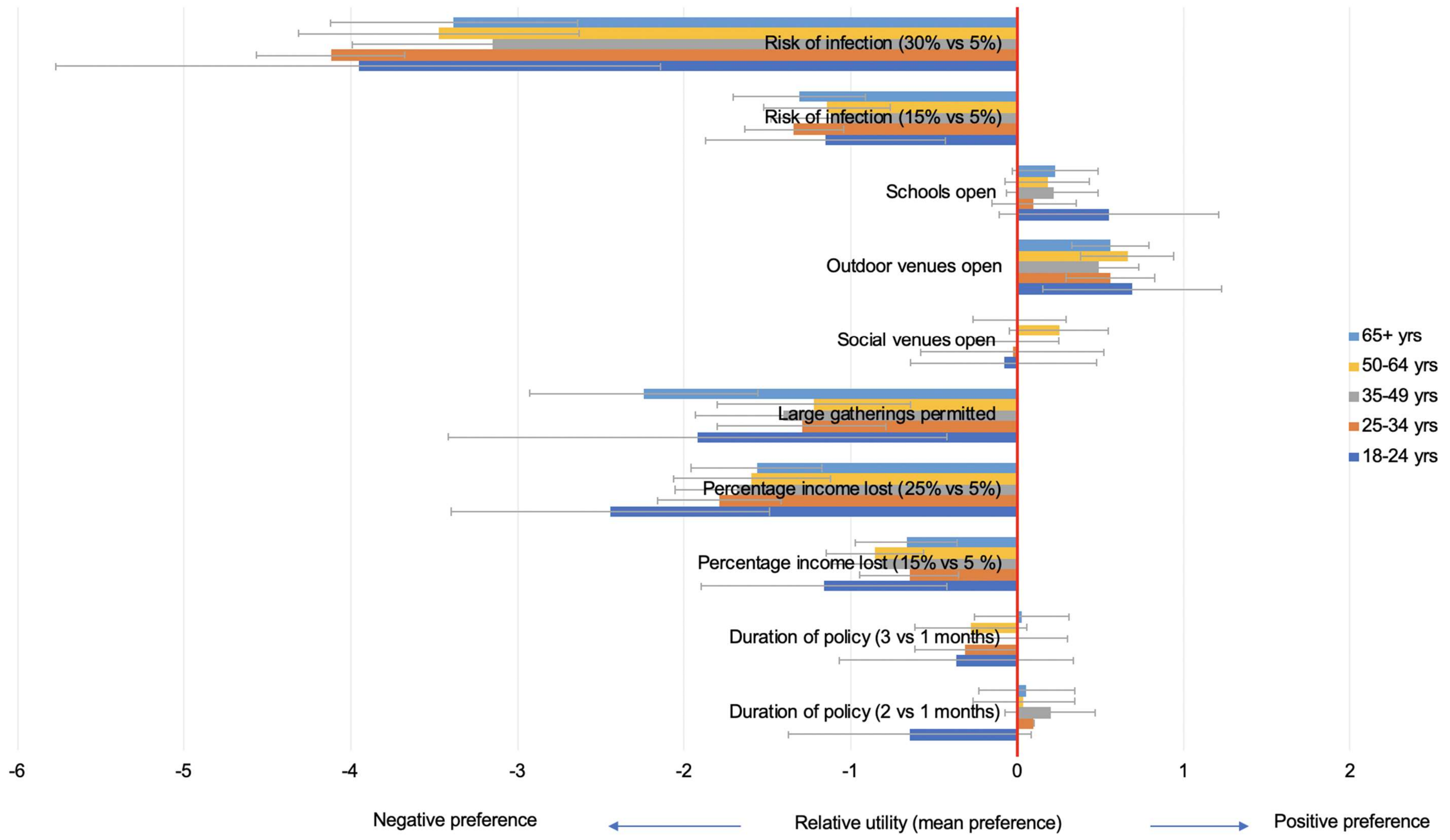

© 2021 Eshun-Wilson I et al. *JAMA Network Open.*

eFigure 2b: Mean preferences by gender

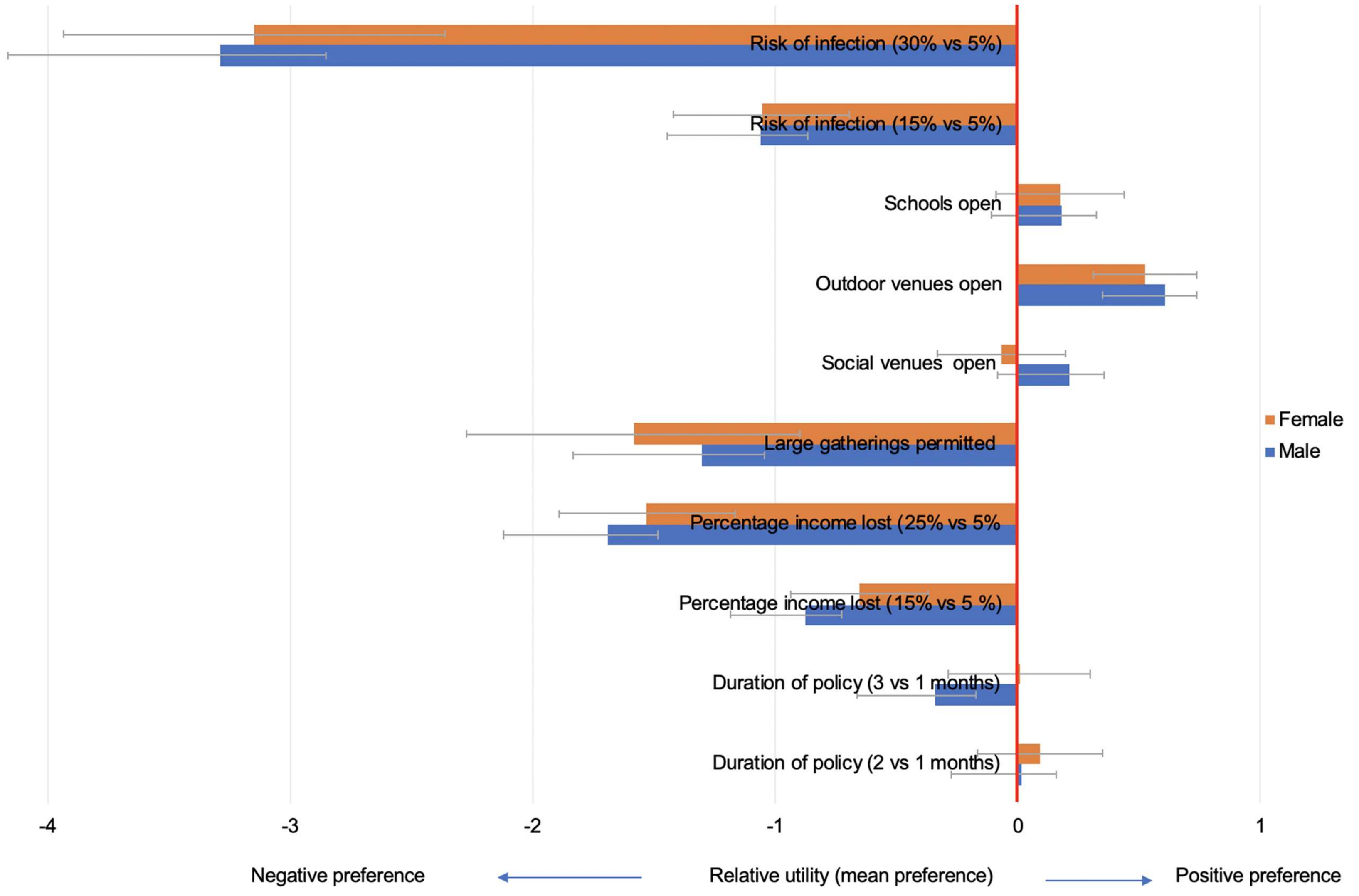

© 2021 Eshun-Wilson I et al. *JAMA Network Open.*

eFigure 2c: Mean preferences by race group

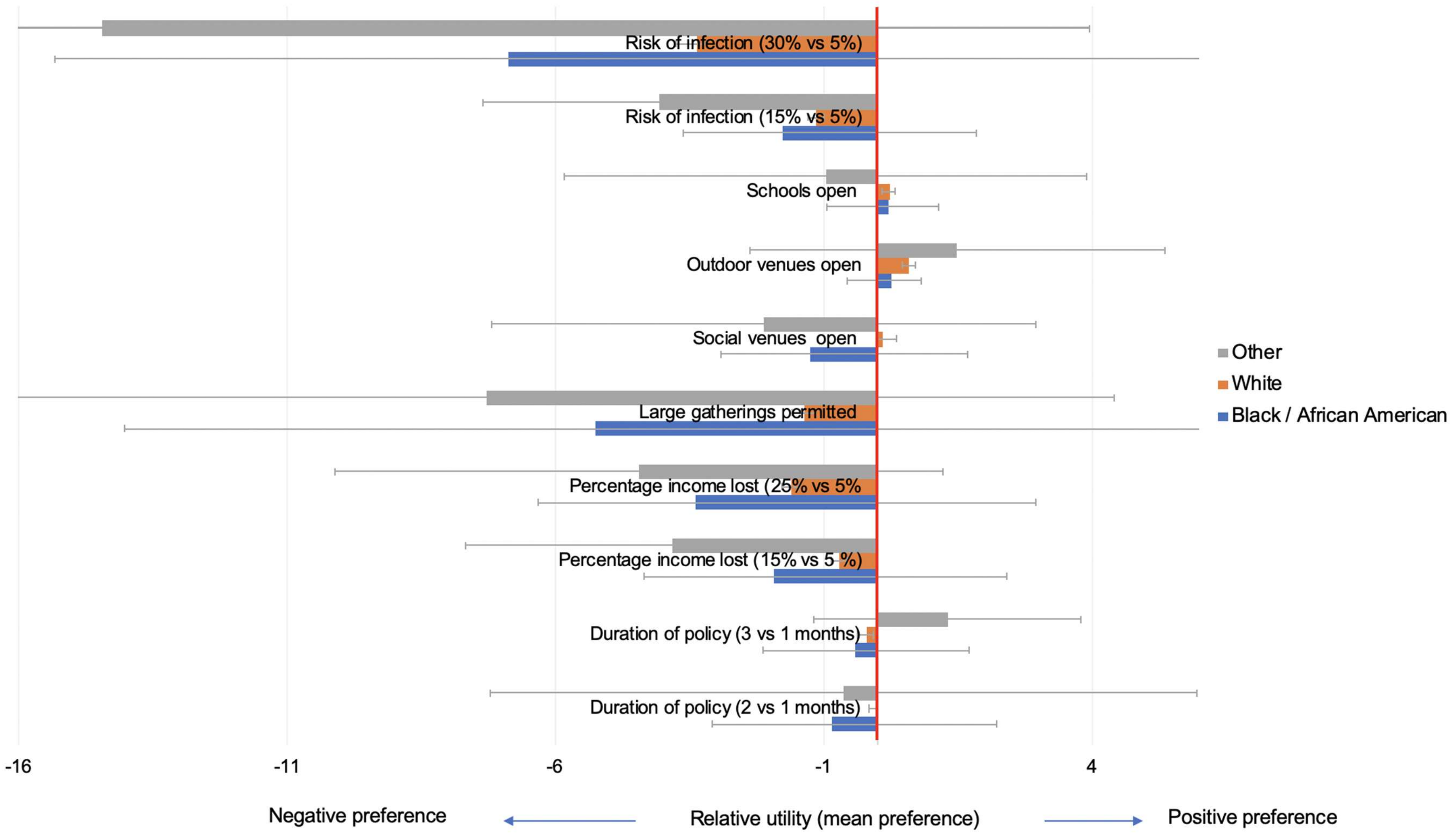

© 2021 Eshun-Wilson I et al. *JAMA Network Open.*

eFigure 2d: Mean preferences by comorbid illness

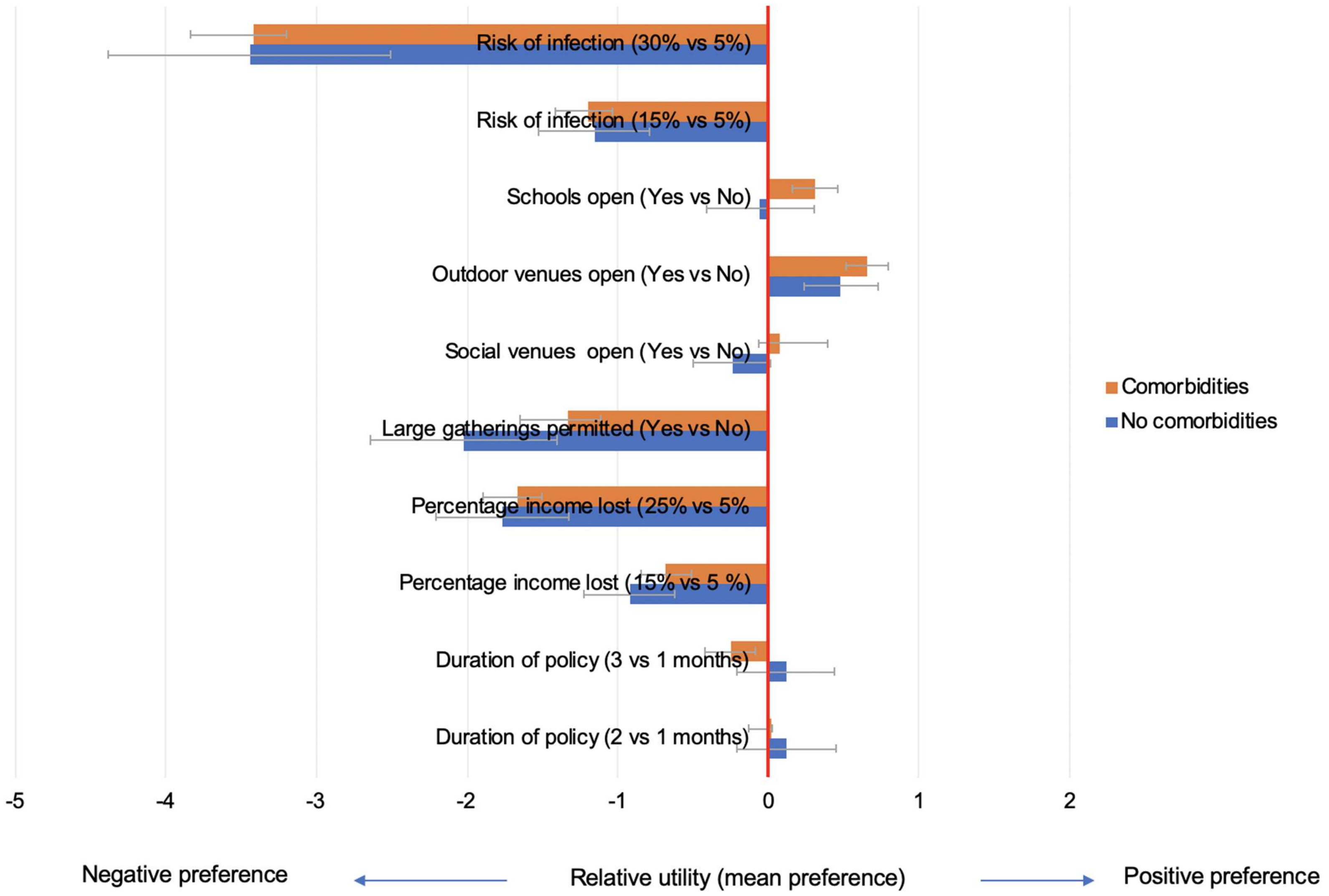

© 2021 Eshun-Wilson I et al. *JAMA Network Open.*

eFigure 2e: Mean preferences by household income

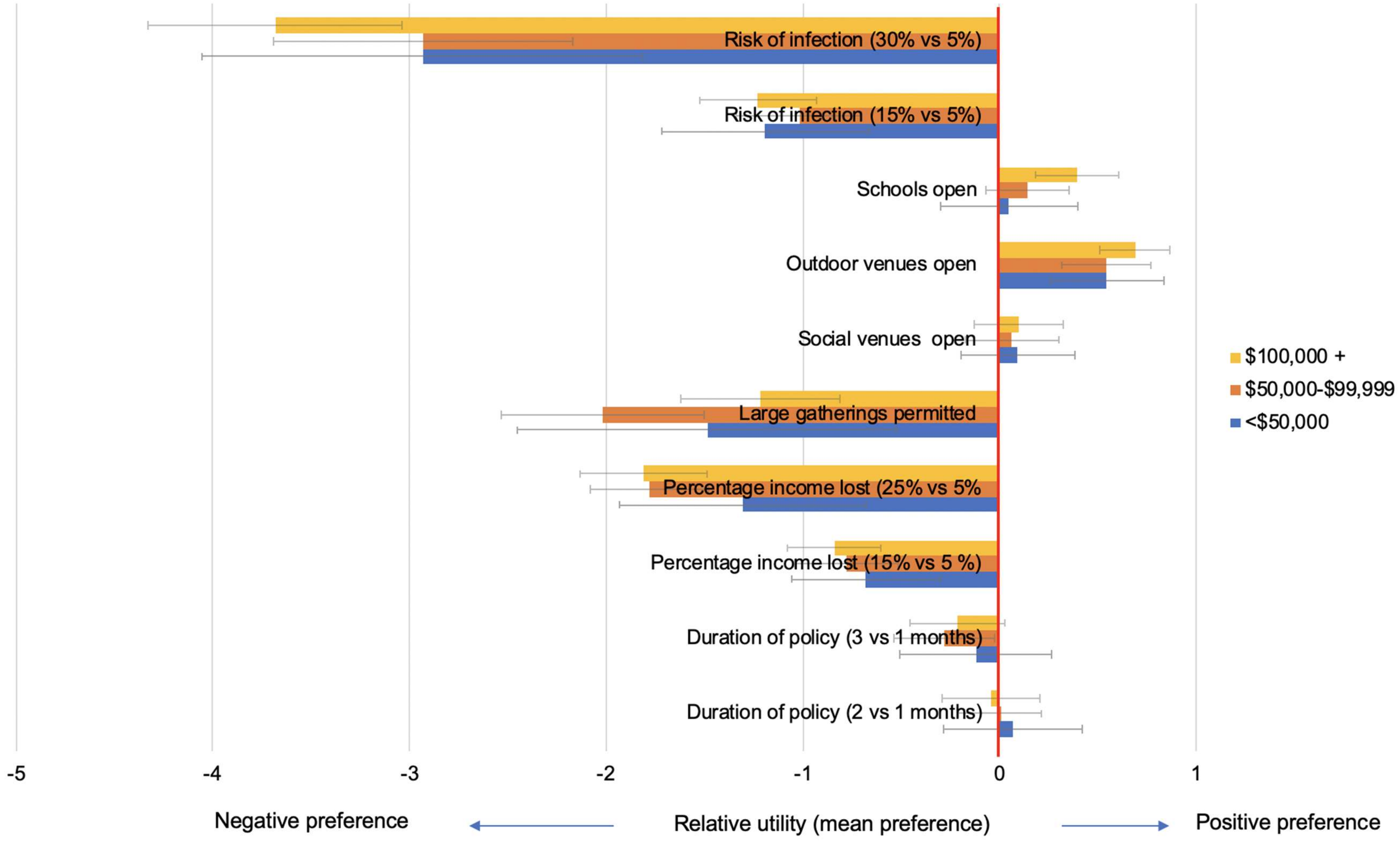

© 2021 Eshun-Wilson I et al. *JAMA Network Open.*

eTable 1. Weighting Strategy

Weighting strata (race, gender, age category)	Missouri population proportion in strata	Number of DCE participants in strata	Proportion of DCE population in strata	Inverse probability weight applied to strata
black_fem_18_24	0.007	5	0.002	3.730
black_fem_25_34	0.010	14	0.006	1.580
black_fem_35_49	0.014	37	0.017	0.843
black_fem_50_64	0.015	34	0.013	1.194
black_fem_65	0.015	17	0.007	1.994
black_male_18_24	0.007	3	0.001	7.717
black_male_25_34	0.010	8	0.004	2.797
black_male_35_49	0.014	10	0.005	2.984
black_male_50_64	0.014	5	0.002	6.174
black_male_65	0.012	2	0.001	12.605
other_fem_18_24	0.005	12	0.005	1.046
other_fem_25_34	0.008	17	0.008	1.004
other_fem_35_49	0.011	18	0.008	1.377
other_fem_50_64	0.012	11	0.004	3.224
other_fem_65	0.011	6	0.002	6.150
other_male_18_24	0.005	8	0.003	1.700
other_male_25_34	0.008	9	0.004	1.918
other_male_35_49	0.011	9	0.004	2.557
other_male_50_64	0.011	6	0.003	3.968
other_male_65	0.009	5	0.001	6.480
white_fem_18_24	0.046	64	0.027	1.724
white_fem_25_34	0.068	267	0.120	0.566
white_fem_35_49	0.093	376	0.167	0.561
white_fem_50_64	0.103	462	0.191	0.539
white_fem_65	0.098	325	0.123	0.795
white_male_18_24	0.047	40	0.017	2.723
white_male_25_34	0.069	111	0.049	1.402
white_male_35_49	0.092	119	0.050	1.818
white_male_50_64	0.095	181	0.078	1.210
white_male_65	0.078	191	0.078	0.994

© 2021 Eshun-Wilson I et al. *JAMA Network Open.*

eTable 2. Characteristics of Those Who Did Not Complete Survey

eTable 2a: Demographic characteristics (completers vs non-completers)

Demographic factor		Completed survey (N=2428)	Not completed (N=617)
Age	18-24yrs	126 (6%)	17 (4%)
	25-34yrs	424 (19%)	52 (13%)
	35-49yrs	553 (25%)	65 (17%)
	50-64yrs	647 (29%)	133 (34%)
	65yrs+	469 (21%)	125 (32%)
Gender	Male	667 (30%)	130 (34%)
	Female	1536 (69%)	248 (65%)
	Non-conforming/other	12 (1%)	3 (1%)
	No Answer	4 (<1%)	1 (<1%)
Race	Black	127 (6%)	23 (6%)
	White	1973 (89%)	336 (89%)
	other	92 (4%)	12 (3%)
	No answer	27 (1%)	7 (2%)
Comorbidities	No comorbidities	1535 (69%)	245 (69%)
	Respiratory comorbidities	320 (14%)	39 (7%)
	Other comorbidities	431 (19%)	78 (14%)
	No answer	11 (<1%)	196 (36%)
Income	< $20,000	97 (4%)	23 (6%)
	$20,000-$49,000	383 (17%)	72 (20%)
	50,000-$99,000	871 (39%)	150 (41%)
	$100,000 +	868 (39%)	117 (32%)
	No answer	209 (9%)	69 (16%)

© 2021 Eshun-Wilson I et al. *JAMA Network Open.*

eTable 2b: Point of survey / DCE termination for non-completers (N=617)

**Point of termination**	**N (%)**
Survey: Introduction	131(21%)
Survey: Age	36 (6%)
Survey: Gender	3 (<1%)
Survey: Income	15 (2%)
Survey: Race	5 (1%)
Survey: Chronic Health Condition	6 (1%)
Survey: Location	5 (1%)
DCE intro	14 (2%)
DCE: Q1	172 (28%)
DCE: Q2	102 (17%)
DCE: Q3	55 (9%)
DCE: Q4	43 (7%)
DCE: Q5	19 (3%)
DCE: Q6	11 (2%)

© 2021 Eshun-Wilson I et al. *JAMA Network Open.*

eTable 3. Mean Preferences and Main Model Selection

eTable 3a: Mean preferences for social distancing policy features

Attribute	Mean preferences				Standard deviation (SD)			
	β	**Low CI**	**High CI**	**p-value**	**SD**	**Low CI**	**High CI**	**p-value**
Duration: 2 vs 1 months	0.00	-0.13	0.12	0.949	-0.09	-0.25	0.07	0.277
Duration: 3 vs 1 months	-0.16	-0.31	-0.02	0.031	0.31	-1.18	1.79	0.687
Income loss: 15% vs 5%	-0.72	-0.86	-0.57	<0.001	-0.04	-0.25	0.17	0.721
Income loss: 25% vs 5%	-1.49	-1.70	-1.29	<0.001	-0.51	-1.13	0.12	0.111
Large gatherings permitted	-1.43	-1.67	-1.18	<0.001	2.62	2.14	3.09	<0.001
Social venues open	0.05	-0.08	0.17	0.451	1.01	0.76	1.27	<0.001
Outdoor venues open	0.50	0.39	0.61	<0.001	-0.25	-0.74	0.25	0.330
Schools open	0.18	0.05	0.30	0.005	-1.13	-1.41	-0.85	<0.001
Risk of infection 15% vs 5%	-1.02	-1.19	-0.84	<0.001	0.06	-0.11	0.23	0.522
Risk of infection 30% vs 5%	-2.89	-3.23	-2.54	<0.001	-0.96	-2.00	0.07	0.069

eTable 3b: Model selection for main preference model

	Degrees of freedom	Log likelihood	AIC	BIC
Model 1: conditional logit - model (no random parameters)	10	-5938.21	11896.42	11978.14
*Model 2: mixed logit model all variables categorical and all attributes fit as random parameters	20	-5261.14	10562.28	10.725.72
Model 3: mixed logit model -Model 2 and duration fit as continuous instead of categorical	18	-5308.35	10652.7	10799.79
Model 4: mixed logit model -Model 2 and income fit as continuous instead of categorical	18	-5307.66	10651.32	10798.41

*Model 1 selected as final model. **Akaike's information criterion. ***Bayesian information criterion

© 2021 Eshun-Wilson I et al. *JAMA Network Open.*

eTable 4. Subgroup Analyses Mean Preferences

		Duration of policy (2 vs 1 months)	Duration of policy (3 vs 1 months)	Large gatherings permitted	Social venues open	Outdoor venues open	Schools open	Risk of infection (15% vs 5%)	Risk of infection (30% vs 5%)	Percentage income lost (15% vs 5 %)	Percentage income lost (25% vs 5%)
Gender	Male	0.01	-0.34	-1.31	0.21	0.61	0.18	-1.06	-3.29	-0.88	-1.70
	Female	0.09	0.01	-1.59	-0.07	0.52	0.17	-1.06	-3.15	-0.66	-1.53
Age	18-24 yrs	-0.64	-0.36	-1.92	-0.08	0.69	0.55	-1.15	-3.96	-1.16	-2.44
	25-34 yrs	0.10	-0.31	-1.29	-0.03	0.56	0.10	-1.34	-4.12	-0.65	-1.79
	35-49 yrs	0.20	0.00	-1.41	0.00	0.49	0.22	-1.11	-3.15	-0.82	-1.69
	50-64 yrs	0.04	-0.28	-1.22	0.25	0.66	0.18	-1.14	-3.47	-0.85	-1.59
	65+ yrs	0.06	0.03	-2.24	0.01	0.56	0.23	-1.31	-3.38	-0.67	-1.56
Race	White	-0.02	-0.21	-1.35	0.10	0.59	0.22	-1.13	-3.37	-0.72	-1.61
	Black	-0.85	-0.40	-5.25	-1.24	0.27	0.21	-1.76	-6.87	-1.94	-3.39
	Other	-0.62	1.31	-7.28	-2.13	1.49	-0.97	-4.06	-14.46	-3.83	-4.44
Annual household income	< $49,999	0.07	-0.12	-1.49	0.09	0.54	0.05	-1.19	-2.93	-0.68	-1.31
	$50,000-$99,999	0.00	-0.28	-2.02	0.06	0.54	0.14	-1.02	-2.93	-0.78	-1.78
	S100,000 +	-0.04	-0.21	-1.22	0.10	0.69	0.39	-1.23	-3.68	-0.84	-1.81
Comorbid illness	Present	0.12	0.12	-2.02	-0.24	0.48	-0.05	-1.16	-3.44	-0.92	-1.77
	None	0.02	-0.24	-1.33	0.08	0.65	0.31	-1.19	-3.42	-0.69	-1.67

Footnotes: Green represents negative preferences and red represents positive preferences; full data for sub-group analyses are presented in Appendix 3; there was no statistically significant difference between utilities between subgroups for all sub-group analyses.

© 2021 Eshun-Wilson I et al. *JAMA Network Open.*

eTable 5. Latent Class Mean Preferences and Model Selection

eTable 5a: Utilities by latent class membership

Levels	Prosocial (14.9%)			Back to normal (13.7%)			Risk averse (48.9%)			Conflicted (22.5%)		
	Utility	95% CI		Utility	95% CI		Utility	95% CI		Utility	95% CI	
Duration: 2 vs 1 months	1.26	0.68	1.84	-0.33	-0.66	0.01	0.08	-0.17	0.33	-0.07	-0.30	0.16
Duration: 3 vs 1 months	1.67	0.98	2.36	-0.90	-1.30	-0.50	0.13	-0.12	0.39	-0.52	-0.80	-0.24
Income loss: 10% vs 5%	-1.04	-1.51	-0.56	-1.45	-1.90	-1.00	-0.80	-1.08	-0.52	-0.75	-1.01	-0.49
Income loss: 25% vs 5%	-2.12	-2.84	-1.40	-2.45	-3.17	-1.73	-1.99	-2.34	-1.65	-2.03	-2.43	-1.63
Large gatherings: permitted vs not	-2.83	-3.87	-1.80	2.19	1.50	2.87	-2.78	-3.30	-2.27	0.22	-0.17	0.61
Social venues open vs closed	-1.73	-2.31	-1.16	1.55	0.99	2.11	-0.69	-0.90	-0.47	0.46	0.26	0.66
Outdoor venues open vs closed	-0.10	-0.56	0.35	1.58	1.07	2.10	0.55	0.34	0.77	0.60	0.39	0.80
Schools open vs closed	-2.71	-3.39	-2.04	1.38	0.93	1.84	-0.43	-0.63	-0.23	1.33	1.06	1.59
Risk of infection 15% vs 5%	0.23	-0.41	0.87	-0.56	-1.07	-0.05	-3.33	-3.87	-2.80	-0.68	-0.99	-0.36
Risk of infection 30% vs 5%	-0.53	-1.37	0.32	-0.69	-1.46	0.09	-7.77	-8.93	-6.62	-3.20	-3.84	-2.57

© 2021 Eshun-Wilson I et al. *JAMA Network Open.*

eTable 5b: Latent class model selection

Model estimated via EM algorithm			
	Log-likelihood	AIC	BIC
2 Classes	-5254.3042	10550.608	10670.037
3 Classes	-5180.6639	10425.328	10607.314
4 Classes	-5114.9636	10315.927	10560.472
5 Classes*	-5070.006	10248.012	10555.114

* Final selected latent class model
AIC=Akaike's information criterion. BIC=Bayesian information criterion

© 2021 Eshun-Wilson I et al. *JAMA Network Open.*

eTable 6. Factors Associated With Latent Class Membership

Characteristic		Conflicted (22.5%)				Pro-social (14.9%)				Back to normal (13.7%)			
		RRR	95% CI		p-value	RRR	95% CI		p-value	RRR	95% CI		p-value
**Gender**	**Female**	1.00			0.455	1.00			0.660	1.00			<0.001
	**Male**	1.12	0.84	1.49		1.09	0.75	1.58		2.19	1.54	3.12	
**Age**	**18-24yrs**	1.00			0.317	1.00			0.674	1.00			0.057
	**25-34yrs**	0.98	0.54	1.78		1.37	0.63	2.94		0.60	0.28	1.27	
	**35-49yrs**	0.68	0.37	1.25		1.60	0.75	3.40		0.84	0.40	1.77	
	**50-64yrs**	0.87	0.48	1.56		1.58	0.69	3.58		1.23	0.57	2.65	
	**65yrs +**	0.71	0.38	1.33		1.23	0.57	2.65		0.65	0.29	1.43	
**Income**	**< $50,000**	1.00			0.102	1.00			0.123	1.00			0.887
	**50,000 < 99,000**	1.41	0.93	2.14		0.69	0.43	1.11		0.96	0.58	1.61	
	**$100,000 +**	1.49	0.99	2.25		0.70	0.44	1.10		0.93	0.56	1.55	
**Race**	**White**	1.00			0.679	1.00			0.333	1.00			0.628
	**Black**	0.88	0.44	1.76		0.77	0.34	1.72		0.58	0.18	1.82	
	**Other**	0.76	0.38	1.49		1.57	0.80	3.10		1.09	0.48	2.48	
**Comorbidity**	**No**	1.00			0.066	1.00			0.478	1.00			0.194
	**Yes**	0.74	0.53	1.02		1.14	0.79	1.64		0.74	0.47	1.16	

Multinomial logisitic regression model, with baseline comparison group set to the risk averse.

© 2021 Eshun-Wilson I et al. *JAMA Network Open.*

References.

1. Orme B. Getting Started with Conjoint Analysis: Strategies for Product Design and Pricing Research. Second Edition ed. Madison, Wisconsin, USA: Research Publishers LLC; 2010.
2. Bridges JF, Hauber AB, Marshall D, Lloyd A, Prosser LA, Regier DA, et al. Conjoint analysis applications in health--a checklist: a report of the ISPOR Good Research Practices for Conjoint Analysis Task Force. Value in health : the journal of the International Society for Pharmacoeconomics and Outcomes Research. 2011;14(4):403-13.
3. Reed Johnson F, Lancsar E, Marshall D, Kilambi V, Muhlbacher A, Regier DA, et al. Constructing experimental designs for discrete-choice experiments: report of the ISPOR Conjoint Analysis Experimental Design Good Research Practices Task Force. Value in health : the journal of the International Society for Pharmacoeconomics and Outcomes Research. 2013;16(1):3-13.
4. Missouri Census Data Center: Population estimates by age 2019 [Available from: http://mcdc.missouri.edu/applications/population/by-age/.
5. United States Census Bureau: Missouri 2019 [Available from: https://www.census.gov/quickfacts/fact/table/MO/PST045219.
6. Lancsar E, Louviere J. Conducting discrete choice experiments to inform healthcare decision making: a user's guide. Pharmacoeconomics. 2008;26(8):661-77.
7. Hole AR. Fitting mixed logit models by using maximum simulated likelihood. Stata Journal. 2007;7(3):388-401.
8. Kleinman L ME, Ryna M, Schmier J, Crawley J, Locke R, et al. Willingness to pay for complete symptom relief of gastroesophageal reflux disease. Arch Intern Med. 2002;162:1361-6.
9. Train KE. EM Algorithms for nonparametric estimation of mixing distributions. Journal of Choice Modelling. 2008;1(1):40-69.
10. Mori M, Krumholz HM, Allore HG. Using Latent Class Analysis to Identify Hidden Clinical Phenotypes. JAMA. 2020;324(7):700-1.

© 2021 Eshun-Wilson I et al. *JAMA Network Open.*
